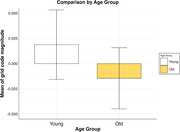# Failure to replicate detection of grid‐cell like representations in human fMRI

**DOI:** 10.1002/alz.086080

**Published:** 2025-01-03

**Authors:** Jonas Kransberg, Sneve H Markus, Emilie Sogn Falch, Anne Cecilie Sjøli Bråthen, Anders M Fjell, Kristine B Walhovd

**Affiliations:** ^1^ LCBC, University of Oslo, Oslo Norway; ^2^ University of Oslo, Oslo Norway

## Abstract

**Background:**

Grid cells are spatially modulated cells in the entorhinal cortex (EC) that fire in a hexagonally patterned grid which tiles the environment. These cells are assumed important in human spatial navigation. The EC is vulnerable to neurodegenerative processes in both normal aging and Alzheimer’s disease and decline in grid cell function may be a key factor in understanding age‐related navigational decline.

Recent work suggests that conjunctive grid and head direction cells can allow for the detection of grid‐like activity based on movement direction. If moving in alignment with a hexagonal grid, EC activation should be higher than movement not aligned with the grid. The present study attempts replicate findings from previous studies of grid‐like signals detected through fMRI.

**Methods:**

The sample included 64 (40 female, 24 male) adults, ages ranging from 18 to 78 (M = 37.67). Participants were subject to a fMRI grid cell paradigm, tasked to passively navigate through a room and remember the location of objects. The movement directions in the virtual environment were used to find the mean grid orientation for each participant set through a general linear model with the sixfold pattern as a regressor, and this was used as a regressor in a second GLM to calculate the grid magnitude, a measure of the stability of the grid codes.

**Results:**

A one‐sample t‐test showed no significant grid code magnitudes (M = 0.002, SD = 0.091) compared to zero for a sixfold symmetry t(63) = 0.19, p = .85. For age‐related analysis, the sample was divided into a younger and older sample through a median split using the median of 30.5 years. A Two Sample t‐test did not show any difference between the younger (M = 0.021, SD = 0.089) and older (M = ‐0.017, SD = 0.090) groups, t(61.99) = ‐1.68, p = .097. Figure 1 shows the mean grid code magnitudes for both age groups.

**Conclusion:**

Preliminary results suggest no evidence for stable grid‐like representations in the present sample. The present study is reasonably powered relative to the previous study, and possible reasons and limitations hindering identification of grid‐like representations should be discussed and further researched.